# Reassessing operability in T4b oral cavity squamous cell carcinoma: a comparative analysis of radiological classification and surgeons' perspective

**DOI:** 10.3332/ecancer.2025.1866

**Published:** 2025-03-06

**Authors:** Fizza Asif Qureshi, Nabeel Humayun Hassan, Rahila Usman, Shafqat Ali Sheikh, Bushra Ayub, Sumra Sattar

**Affiliations:** 1 Department of Otorhinolaryngology Head and Neck Surgery, Patel Hospital, Karachi 75300, Pakistan; 2 Head of Radiology Department, Chughtai Health Care, Karachi 54000, Pakistan; 3 Department of Seminar, Patel Hospital, Karachi 75300, Pakistan

**Keywords:** oral cavity cancer, advanced stage, mandibular notch, diagnostic imaging, T4b, AJCC staging

## Abstract

**Introduction:**

Oral cavity squamous cell carcinoma (OCSCC), being more prevalent in developing countries due to the intake of chewable tobacco, is treatable if patients present at an early stage. According to the American Joint Committee on Cancer, T4b disease is termed inoperable and treated with palliative intent. However, with the passage of time, these have been operated on, with comparable disease-free survival rates with T4a disease. On radiological investigation, the mandibular notch is a structure dividing the infratemporal fossa into two. The objective is to determine the frequency of supra-notch and infra-notch T4b OSCC tumours and to compare them with surgeons’ perspective in terms of operability.

**Methods:**

T4b staged patients of OCSCC were retrospectively reviewed along with the computed tomography (CT) scans to term them as infra-notch and supra-notch cases. They were then reviewed by two surgeons for surgical intervention as the treatment option and these two decisions were evaluated for agreement.

**Results:**

Of the 51 patients included, 36 were primary for buccal mucosa. According to the radiologist, 30 were infra-notch and the remaining 21 were supra-notch diseases. The first surgeon deemed 33 cases as operable and 18 as inoperable, whereas the second surgeon labelled 30 as operable and 21 as inoperable. For infra-notch cases, the first surgeon’s opinion was operability in 27 and the second surgeon’s opinion was operability in 24 cases. For supra-notch cases, the results were similar as both deemed 71.4% as inoperable i.e., 15 out of 21 patients. The agreement between radiologist and first surgeon, analysed by Cohen’s Kappa, was 0.514, which is a moderate agreement, and between radiologist and second surgeon was 0.628, which shows substantial agreement.

**Conclusion:**

Therefore, we conclude that mandibular notch can be used as a landmark to classify supra-notch and infra-notch tumours. Supra-notch tumours are most likely inoperable.

## Introduction

The overall burden of cancers and related mortality is rapidly increasing throughout the world. According to the Globocon 2020, WHO estimates that there will be 28.4 million new cases by 2040, an estimated 47% rise compared to 2020. The lesser chewing habits and geographic heterogeneity are credited with this overall global decrease in its prevalence; however, it is still the most prevalent cancer in South Asia, South Central Asia and the Pacific Islands (Papua New Guinea has the highest incidence rate globally in both sexes) [1]. According to it, lip and oral cavity cancers are the second most common malignancy for females, while they are the most common malignancy in males in Pakistan [2].

Patients usually present at a locally advanced stage of T4a or T4b (>50%) [3]. These cases are a big treatment challenge. One of the reasons is that they have poor outcomes with a median survival of 6–9 months [4]. However, carefully selected patients who had surgical treatment may have a better prognosis with median survival as high as 19.6 months [5]. According to the American Joint Committee on Cancer (AJCC) 2023 and National Comprehensive Cancer Network (NCCN) guidelines, the advanced stage of T4b has a poor outcome, because of the high rate of local recurrence [6, 7].

It is challenging to determine on radiological imaging whether infratemporal fossa (ITF) is involved or not [8, 9]. In addition, it is challenging to appropriately stage the disease and determine the extent of spread owing to the limited clinical examination caused by trismus [10].

In the last few decades, the treatment modality has changed from the palliative intent, and surgeons have gone towards surgical excision. There have been studies like Pillai *et al* [11] who compared oncological outcomes between pT4a and pT4b oral cavity squamous cell carcinoma (OCSCC) treated with surgery and then concurrent chemoradiation therapy (CCRT), and the results were comparable and similar. A study by Mohiyuddin *et al* [12] stated the 2-year overall survival (OS) of T4b patients treated with primary surgery followed by radiotherapy ± chemotherapy, to be 60%. Another study conducted by Kang *et al* [13] concluded that there was a significant difference when comparing 5-year OS outcomes of surgically treated T4a and T4b OCSCC. Another study by Patel *et al* [14] also determined that clinical T4a and T4b patients who underwent surgery with CCRT showed no difference in the 2-year survival rate.

The mandibular notch is a semilunar depression separating the cricoid and condylar processes of the mandible, through which recently, a further division of the T4b stage has been proposed in some intervention and outcome studies. This has been reviewed in the study by CT Liao *et al* [15] that is there a difference between patients with disease extension until this landmark compared to patients having extension beyond it. They concluded that infra-notch T4b had better 5-year loco-regional control and survival. Mair *et al* [16] evaluated primary surgical resection followed by adjuvant therapy in 135 T4a and 75 infra-notch T4b OCSCC Indian patients. No discernible difference was found in 3-year locoregional control (71.1% versus 61.8%: *p* = 0.107) or OS (49.6% versus 41.1%: = 0.518) when comparing these cases [16]. Much work has been put into finding a curative pathway for T4b disease instead of palliative care and further, to conclude if the current staging criteria and treatment protocol needs changing for this locally aggressive group having close disease-free as well as OS.

Thus, the goal of our study is to determine an objective criterion on imaging for the likelihood of operability of T4b diseases with the mutual decision of the primary surgeon plan and the radiologists.

## Material and methods

This study was conducted retrospectively, employing a cross-sectional design within the Departments of Otorhinolaryngology Head and Neck Surgery, and Radiology at Patel Hospital, the period of which was January 2021–December 2022. Approval for the study was granted by the Hospital’s Ethics Committee, under the reference ERC# PH/IRB/2023/023. The research included patients between 20 and 80 years, diagnosed with OCSCC and categorised as T4b lesions while excluding those who had undergone prior treatment by any modality, exhibited disease recurrence or possessed a history of head and neck surgery. Patients with radiological investigations conducted outside the institute or those with evidence of carotid artery encasement were also excluded.

A thorough review of patients’ files was conducted, recording all relevant demographics and identifying T4b cases. Computed tomography (CT) scans of these cases were assessed by a consultant radiologist to categorise them as supra-notch or infra-notch tumours based on the criteria established by Liao *et al* [7, 15]. The mandibular notch was used as a key landmark on CT scans for this, with a preference for obtaining 1–2-mm axial, coronal and sagittal sections of the CT scan. The division of ITF into supra-notch and infra-notch was determined based on a trans-axial plane passing through the notch between the coronoid and condylar processes.

Furthermore, two surgeons, each with over 5 years of experience, independently reviewed these CT scans to assess the operability of the patients, without prior knowledge of the radiologist’s classification system or each other’s conclusions. Statistical analyses were performed using SPSS version 21. Demographic variables were presented in terms of mean ± standard deviation for quantitative variables (such as age, site of lesion and duration of lesion) or as median with interquartile range (IQR), depending on the normality assumption, which was tested using the Shapiro–Wilk test. The infra-notch/supra-notch variable and operability were presented as frequencies. The agreement between the assessments of the two surgeons was evaluated using the Kappa test, with a *p*-value <0.05 considered significant.

## Results

Table 1 presents the key demographics of the patients. Out of 51 patients included, 42 were males and 9 were females, resulting in a male-to-female ratio of 4.6:1. The median age was 49 years, with an IQR of 45–59 years. Among the 51 patients, 15 individuals were found to have multiple addictions, with 12 of them exhibiting addictions to chewable tobacco. No patients had any addiction history of smoking or alcohol. All patients were confirmed to have OCSCC through biopsy. Of these patients, 36 had disease originating from the buccal mucosa subsite, 6 had disease originating from the alveolus and 9 had disease originating at the retromolar trigone.

**Figure 1.**
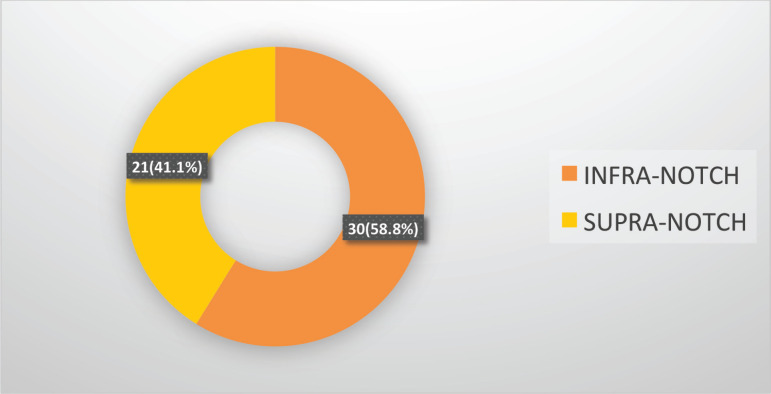
Opinion of the radiologist.

**Figure 2.**
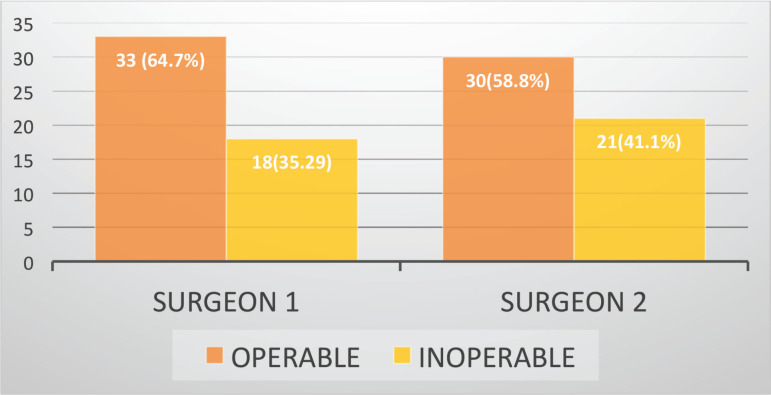
Opinion of the two surgeons regarding operability.

Figure 1 illustrates the radiologist’s assessment based on the CT scans, where 30 (58.8%) patients were identified with infra-notch disease and the remaining 21 (41.7%) were diagnosed with supra-notch disease. Figure 2 depicts the evaluations of the two surgeons, with the first surgeon classifying 33 (64.7%) patients as operable and 18 (35.3%) as inoperable, while the second surgeon categorised 30 (58.8%) patients as operable and 21 (41.2%) as inoperable.

A comparison between the first surgeon’s evaluation and that of the radiologist (as presented in Table 2) reveals that out of the 30 cases labelled as infra-notch, the first surgeon identified 27 as operable and only 3 as inoperable. In contrast, of the 21 supra-notch cases, only 6 were deemed operable and the remaining 15 were considered inoperable. The agreement between the two, evaluated using the Kappa statistic, was found to be 0.514, indicating a moderate level of agreement between the two assessments.

Likewise, in the second surgeon’s assessment compared to the radiologist’s findings (Table 3), it was determined that the second surgeon categorised 24 out of the 30 infra-notch cases as operable and the remaining 6 as inoperable. Similarly, for the 21 supra-notch cases, only 6 were identified as operable, while the remaining 15 were classified as inoperable, aligning with the first surgeon’s assessment.

The agreement between the second surgeon and the radiologist, as calculated using the Kappa statistic, was found to be 0.628, indicating a substantial level of agreement between the two assessments.

According to Table 4, when the decisions of the two surgeons were set side by side, the first surgeon concluded that the decision for operability was similar for 24 cases, and for inoperability, it was 12 cases. Six cases were deemed inoperable by the first surgeon but were decided as operable by the second and, 9 cases that were operable according to the first surgeon were inoperable according to the second.

The level of agreement between the two surgeons was calculated by Kappa to be 0.308, which indicates fair agreement.

We also analysed the subset of patients who proceeded with surgery. For local tumour excision extending to the ITF, the coronoid process of the mandible or the ascending ramus was resected, depending on the degree of mandibular erosion. In some cases, part of the lower pterygoid plates was also removed to ensure oncologic clearance. According to Table 5, all of the patients that were labelled infra-notch were operated on except one that was not operated on due to medical reasons, and among 21 patients with supra-notch diseases, only 9 were operated. The chi-square test applied *p*-value was highly significant indicating a strong association between infra-notch tumours and likelihood of surgery. Consequently, all patients assessed as operable, except one, underwent surgery. None of the patients had neo-adjuvant chemotherapy or radiation as per our protocol, if a patient is not operated is referred for palliative chemotherapy/radiotherapy. Hence, 30 patients were deemed operable by the surgeons depending upon the disease extension. One of them was not operated on due to medical reasons, and the rest of them was operated. The 30 operated patients have infra-notch disease and include 27 and 24 patients deemed operable by the first and second surgeons, respectively.

## Discussion

According to the AJCC in 2020, stage T4b involves the invasion of the masticator space, pterygoid plates, skull base and/or encasement of the internal carotid artery, leading to the reclassification of OCSCC as T4b disease, representing an inoperable tumour [4, 8]. This stage is considered unresectable due to the difficulty in achieving a clear margin, which is recognised as the single most significant prognostic factor [10, 11].

The complexity of T4b disease necessitates its subclassification for better management. Although the term ‘ITF’ is not explicitly outlined in the eighth edition of the AJCC classification, its interchangeable use with the term ‘masticator space’ has been observed. However, various studies, including the work by Pillai *et al* [11] in 2019 and later by Kang *et al* [17], have emphasised the need to re-evaluate and reclassify stage IVb due to the distinct behaviour of T4b and N3b diseases, both of which were initially classified similarly but exhibit different outcomes.

In our study, the surgeons occasionally categorised the disease in the ITF as resectable, while at other times, it was considered unresectable, emphasizing the lack of clear, objective criteria used by surgeons to distinguish operability. This observation aligns with the findings of Trivedi [18] as highlighted in their literature review. Furthermore, the radiologist in our study identified all these tumours as involving the masticator space.

Over the past decade, the term ‘masticator space,’ coined by radiologists to equate to the ITF, has been further divided into supra-notch and infra-notch, utilizing the mandibular notch as a reference point. Van Cann *et al* [19] have clearly outlined this and provided a well-structured algorithm for utilizing this classification system. Similarly, in our study, the radiologist classified the tumours as supra-notch and infra-notch, with 21 (41.4%) supra-notch cases and 30 (58.8%) infra-notch cases. Various previous studies have demonstrated that this criterion can be used to identify tumours that can be resected with better outcomes, as highlighted in a study by Bera and Tripathi [20]. In addition, Bera *et al* [21]’s research revealed no difference in nonsurgical treatment when used in T4b unresectable diseases. In a meta-analysis by Rao *et al* [22] which included 1,190 T4b patients, it was found that infra-notch tumours were resected with better margin control and exhibited similar outcomes to T4a patients.

The disease below the mandibular notch is often deemed resectable, while the supra-notch extension is generally labelled as unresectable due to its proximity to the skull base. It will be difficult to achieve R0 resection in supra-notch tumours because the distance between notch and skull base is only 2 cm as mentioned by Liao *et al* [23]. This is further complicated by the fact that the upper third of the pterygoid plates, lateral pterygoid muscle and the tendon of the temporalis muscle all reside within the supra-notch compartment. Considering these, the decision to operate is determined when a 1-cm margin can be safely achieved, and the tumour does not involve the lateral pterygoid muscle, upper part of the pterygoid plates or the temporalis tendon, as indicated in Bera *et al* [21]’s study. Our study yielded similar results, despite the surgeons’ unfamiliarity with the division of supra-notch and infra-notch tumours. Both surgeons labelled most of the infra-notch tumours as operable and most of the supra-notch tumours as inoperable. Similarly, of the 21 supra-notch tumours, only 6% were labelled operable by both surgeons. The level of agreement between the two surgeons, by Kappa, was found to be 0.308, which indicates fair agreement.

We also evaluated the level of agreement between the surgeons’ and radiologist’s opinions. When comparing surgeon 1 and the radiologist, the Kappa was 0.514, indicating moderate agreement. Similarly, the Kappa was 0.628 between surgeon 2 and the radiologist, demonstrating substantial agreement. The analysis comparing radiologists’ assessment of infra- and supra-notch tumours with surgeons’ evaluations of operability based on disease extent revealed a highly significant association. This underscores the importance of this objective criterion, suggesting its practical value in clinical decision-making. These findings align with the conclusions of Rao *et al* [22]’s meta-analysis, which reported that infra-notch tumours were more likely to be resected with clear margins, making them more operable and associated with improved survival outcomes.

It is essential to acknowledge that this study has several potential limitations. First, the study did not assess surgical outcomes of patients who underwent operations, to determine whether oncological clear margins were achieved in cases labelled as operable or not. Second, the study did not evaluate survival outcomes and recurrence, which is the ultimate goal of treatment. It might be worthwhile to compare the outcomes of both patient groups who underwent operations and those who did not to assess the translational impact of the supra-notch and infra-notch classification in terms of disease-free survival.

However, this study has demonstrated that the use of this classification system can help establish objective criteria for selecting operable patients, while the other parameters described above can be investigated in the future to evaluate the survival benefits of using this classification system.

## List of abbreviations

AJCC, American Joint Committee on Cancer; CCRT, Concurrent chemoradiation therapy; CECT, Contrast-enhanced CT; DSS, Disease-specific survival; HNSCC, Head and neck squamous cell carcinoma; ITF, Infratemporal fossa; MRI, Magnetic resonance imaging; NCCN, National Comprehensive Cancer Network; OCSCC, Oral cavity squamous cell carcinoma; OS, Overall survival.

## Conflicts of interest

The authors do not have any conflicts of interest to declare.

## Funding

No funding was received for this study.

## Author contributions

Fizza Asif Qureshi: Introduction writing, material and method writing, data collection, result writing, manuscript designing and approval of the final version of the manuscript. Nabeel Humayun Hassan: Conception of the study, data collection, data review, discussion writing and approval of the final version of the manuscript. Rahila Usman: Data review, discussion writing and approval of the final version of the manuscript. Shafqat Ali Sheikh: Data review, discussion writing and approval of the final version of the manuscript. Bushra Ayub: Data review, data analysis and approval of the final version of the manuscript. Sumra Sattar: Approval of the final version of the manuscript.

## Figures and Tables

**Table 1.** Patients’ demographics.

		Frequency (percentages)
Comorbid	Hypertension	9 (17.6%)
	Diabetes	12 (23.5%)
	None	24 (47%)
	Multiple	6 (11.7%)
Gender	Male	42 (82.3%)
	Female	9 (17.6%)
Addiction	Pan	12 (23.5%)
	Chalia	12 (23.5%)
	Gutka	12 (23.5%)
	Multiple	15 (29.4%)
Site of lesion	Buccal mucosa	36 (70.5%)
	Alveolus	6 (11.7%)
	Retromolar trigone	9 (17.6%)
	Hard palate	0

**Table 2.** Comparison between the first surgeon and the radiologist’s decision.

		Radiologist’s opinion		Total
		Infra-notch	Supra-notch	
First surgeon's opinion	Operable	27	6	33 (64.7%)
	inoperable	3	15	18 (35.3%)
Total		30 (58.8%)	21 (41.2%)	51

**Table 3.** Comparison between the second surgeon and the radiologist’s decision.

		Radiologist's opinion		Total
		Infra-notch	Supra-notch	
Second surgeon's opinion	Operable	24	6	30 (58.8%)
	inoperable	6	15	21 (41.2%)
Total		30 (58.8%)	21 (41.2%)	51

**Table 4.** Comparison between the decisions of the two surgeons.

		First surgeon’s opinion		Total
		Operable	Inoperable	
Second surgeon’s opinion	Operable	24	6	30 (58.8%)
	Inoperable	9	12	21 (41.2%)
Total		33 (64.7%)	18 (35.3%)	51

**Table 5.** Comparison of radiologist opinion and surgeons’ assessment of operability of disease.

		Surgery status		Total	*p*-value
		Operated	Not operated		
Radiologists Opinion	Infra-notch	29 (96.6%)	1 (3.33%)	30 (58.8%)	0.000
	Supra-notch	9 (42.8%)	12 (57.14%)	21 (41.1%)	
Total		38(74.5%)	13(25.4%)	51	
